# mSphere of Influence: The power of *in situ*—structural biology without leaving “home”

**DOI:** 10.1128/msphere.00798-25

**Published:** 2026-03-09

**Authors:** Nicholas A. Wood

**Affiliations:** 1 Department of Biophysics, Medical College of Wisconsin 5506, Milwaukee, Wisconsin, USA; University of Nebraska Medical Center, College of Medicine, Omaha, Nebraska, USA

**Keywords:** biophysics, structural biology, AlphaFold

## Abstract

Nicholas A. Wood works in the field of biophysics with an emphasis on *in situ* protein structures in microbiological systems. In this mSphere of Influence article, he reflects on the power and limitations of AlphaFold, open questions in structural biology, and the cross-disciplinary potential of microbiological training.

## COMMENTARY

As scientists, we share key similarities with the average 5-year-old child. We are often distracted by things we find intriguing, regardless of situational applicability. We spend countless hours attacking new obstacles until we find something that works. Most importantly, we relentlessly ask the question “why?” despite knowing that the answer will never be truly satisfactory and will demand more questions. I failed to realize that I would have to relearn these skills when I first joined the laboratory of Dr. Scot Ouellette during my undergraduate career at the University of South Dakota, where I probed molecular interactions and the resulting developmental consequences in *Chlamydia trachomatis*. While I struggled immensely in the early years, Dr. Ouellette led by example to instill within his students a deep appreciation for the scientific method. Through a rather fortuitous relocation of the Ouellette lab to the University of Nebraska Medical Center—where I had applied for graduate school—I continued my graduate career with Dr. Ouellette. His trust in my ability afforded me freedom to pursue my interests within the bounds of my project, which allowed me to slowly realize that I had a structural biology itch that I could not quite scratch. My molecular “why?” questions often stalled down the road by a paucity of chlamydial protein structures. However, my training to this point would not allow me simply to accept this. I set out to develop a repository of Phyre2 (1) predictions of every chlamydial ORF. After countless hours of bootstrapping work, I had at last predicted and annotated every single chlamydial protein, including predicted and hypothetical genes, to the best of my ability. Then, AlphaFold2 was released, streamlining these analyses in a way I never thought possible.

The advent of AlphaFold ushered in an extraordinary era of enhanced interest in structural biology due to its unprecedented accuracy, accessibility, and ease of use (2). Seemingly every single protein structure was now within reach. Within months, Uniprot became a go-to repository for predicted structures of virtually every known or hypothetical protein. I spent numerous hours downloading PDB files and launching them in the DALI server (3) simply to see what, up until that point, was completely unknown. While my time as a graduate student neared its end, I carried this excitement with me as I entered a postdoctoral position in the structural realm.

Armed with my microbiology and molecular biology training, I set out to add structural biology as another notch in my belt. To this end, I joined the Department of Biophysics at the Medical College of Wisconsin in the laboratory of Dr. Francesca Marassi, who specializes in biophysical structural techniques, including both solution and solid-state nuclear magnetic resonance (NMR). However, rather than setting the structural world ablaze with my integrative approach to hypothesis testing, I ran face-first into a massive knowledge barrier for which my self-training in AlphaFold had, rather unsurprisingly, failed to prepare me. While we still utilize AlphaFold as another invaluable tool for our work, I quickly learned that AlphaFold structural predictions are just that—predictions. A high-confidence AlphaFold prediction serves as a robust starting point for hypothesis generation but cannot substitute for rigorous experimentation. Proteins are not simply static masses of pipes, ribbons, and helices, despite how much I wanted this to be true for the sake of simplicity. Even highly stable proteins experience molecular fluctuation in response to a multitude of internal and external factors. True dissection of protein function requires a massive body of experimentation, including structural (e.g., NMR, crystallography, cryo-EM), computational (e.g., molecular dynamics simulations), and biological work. While this experimentation is obviously the backbone of the scientific process, I still find that I must continuously remind myself that the AlphaFold predictions are neither dogmatic nor unreliable. Rather, AlphaFold is a tool that has cemented itself as an irreplaceable component of the structural biology arsenal and can serve as a complementary approach to classic methodologies.

Given that my formal training is in microbiology and that my ability to enter amino acid sequences into AlphaFold is not enough for a complete project, I next had to ask myself where I fit into the structural biology equation. While I lacked the answer on day 1, cursory scans of solved protein structures in the PDB continuously revealed one critical factor that is missing from a majority of entries. What could possibly be missing from so many rigorously validated structures in such a highly curated database? The answer lies in one of the major questions often wrought from *in vitro* studies: do the data recapitulate function in the native context? While the importance of structures of purified recombinant proteins cannot be understated, I found myself asking, “What if we did not have to extract a protein from the very environment in which it is evolutionarily designed to function?” Fortunately for me, my new PI had forged a career in which one main emphasis is studying proteins *in situ*. Even more fortunately for me, her scientific curiosities include microbiology. While we are not the first group to delve into *in situ* biology, the current progress would not have been possible without the microbiological training I have received up to this point. Together, we have initiated solid-state NMR studies to dissect the atomic mechanism of protein-driven outer membrane vesiculation in *Salmonella* without extracting the client protein from the membrane. These studies, combined with the AlphaFold prediction, guide molecular dynamics simulations to permit visualization in as close to a native environment as possible.

Despite the challenges of entering a completely new field, I know that this path is the correct one. I aim to continue leveraging *in situ* structural biology throughout my career to ask how—rather than if—we can “see” a protein function in its natural habitat. While I still have a long road of learning ahead of me, I hope that my journey resonates with other microbiologists who may hold interests in other fields. In today’s highly collaborative scientific landscape, taking that leap into a new area does not require renouncing one’s microbiological roots. Rather, embracing microbiology expertise while training with experts in other fields supports the development of novel methodologies and insights that can be used in the relentless pursuit to answer that question of “why?”.

## References

[1] Kelley LA, Mezulis S, Yates CM, Wass MN, Sternberg MJE. 2015. The Phyre2 web portal for protein modeling, prediction and analysis. Nat Protoc 10:845–858. doi:10.1038/nprot.2015.053 25950237 PMC5298202

[2] Jumper J, Evans R, Pritzel A, Green T, Figurnov M, Ronneberger O, Tunyasuvunakool K, Bates R, Žídek A, Potapenko A, et al.. 2021. Highly accurate protein structure prediction with AlphaFold. Nature 596:583–589. doi:10.1038/s41586-021-03819-2 34265844 PMC8371605

[3] Holm L. 2022. Dali server: structural unification of protein families. Nucleic Acids Res 50:W210–W215. doi:10.1093/nar/gkac387 35610055 PMC9252788

